# Cracking the code: a comparative approach to plant communication

**DOI:** 10.1080/19420889.2021.1956719

**Published:** 2021-08-17

**Authors:** Bianca Bonato, Francesca Peressotti, Silvia Guerra, Qiuran Wang, Umberto Castiello

**Affiliations:** a Dipartimento di Psicologia Generale, Università Degli Studi di Padova, Padova, Italy; b Dipartimento di Psicologia dello Sviluppo E della Socializzazione, Università degli Studi di Padova, Padova, Italy

**Keywords:** Language, plants, cognition, ecology, plant behavior

## Abstract

The linguistic behavior of humans is usually considered the point of reference for studying the origin and evolution of language. As commonly defined, language is a form of communication between human beings; many have argued that it is unique to humans as there is no apparent equivalent for it in non-human organisms. How language is used as a means of communication is examined in this essay from a biological perspective positing that it is effectively and meaningfully used by non-human organisms and, more specifically, by plants. We set out to draw parallels between some aspects characterizing human language and the chemical communication that occurs between plants. The essay examines the similarities in ways of communicating linked to three properties of language: its combinatorial structure, meaning-making activities and the existence of dialects. In accordance with the findings of researchers who have demonstrated that plants do indeed communicate with one another and with organisms in their environment, the essay concludes with the appeal for an interdisciplinary approach conceptualizing a broader ecological definition of language and a constructive dialogue between the biological sciences and the humanities.

## Introduction

The role that cognition plays in shaping behavior in the animal realm has been receiving increasing attention over the last thirty years [1,2]. Given its traditional significance in human psychology, the modern study of cognition assumes to a greater or lesser extent that human cognitive abilities constitute the standard template for any theorizing within this area of research [3]. Independently of and beyond the anthropocentric tendencies that continue to exist, we are convinced that cognition needs to be approached from a wider biological perspective. It is now abundantly evident that plant behavior, which is more sophisticated than previously thought [4], reflects complex, flexible cognitive processes [1]. We are persuaded that the scientific community needs to spend efforts systematically investigating plant cognition, which shows unmistakable parallelisms with some forms of cognitive processing used by non-human animals, such as those underlying communicative processes. Although the idea that plants communicate may seem strange to the general public, numerous professionals from a host of scientific fields are genuinely awestruck by the complexity of plant responses, that is, by plants’ ability to interact and adapt to an ever-changing environment.

This essay is concerned with language and communication in human and non-human organisms and, in particular, in plants. The first part is dedicated to providing a brief overview of language and forms of communication that exist in human and non-human organisms focusing on properties such as meaning-making activities, combinatorial structures and the existence of dialect. The second part concentrates on studies providing empirical evidence of plants’ ability to recognize kin from stranger and to communicate. The subsequent one examines the specific rules governing the chemical molecules used as cues in plants’ communication processes. It then goes on to discuss the importance of studying the communication between plants from a wider perspective and highlights the vulnerability of the chemical communication process within the context of the global climate change. Finally, all these pieces are filed under a comparative heading in the attempt to link some already understood aspects of human language with novel ones related to plant communication.

The underlying aim of this essay is to advance the theory that what goes on between plants and non-human organisms can be considered a communication process or biocommunication conveyed by signs and governed by specific rules [5]. We will argue that the similarities between the forms of communication that plants use and those utilized by human and non-human organisms deserve further attention as they may lead us to unlocking the code that plants use to communicate.

## A brief overview of human and non-human language

Discussions on language across taxa are necessarily conditioned by how we define “language” and the fact that it is for the most part considered unique to human beings [6]. Human language has been described as a complex system of signs that arbitrarily map into meanings [7, see also 8–10]. The system is characterized by a hierarchical structure whereby minimal units at one layer are combined following grammatical rules to form larger units at the next one. For example, phonemes are combined to form morphemes, morphemes are combined to form words and words are combined to form sentences. This combinatorial aspect is characterized by productivity and creativity, meaning that every utterance is – in principle – unique and its production (or comprehension) is intrinsically related to the semantic, syntactic and pragmatic context within which it is generated.

The emergence of language is considered a crucial occurrence in the evolution of human history, an innovation that radically changed the character of human interactions [11]. Attempts to shed light on the evolution of human language have been undertaken by investigators working in a host of research domains investigating: the social behavioral characteristics of primates [12,13], the unity and diversity of human language [14,15], the development of language in children [16], and the genetic and anatomical correlates of language expertise [17,18]. It would seem, in any case, that at some point millions of years ago language began evolving when hominids started using sounds to share meanings and thoughts with one another [11] and establish social interactions.

According to a traditional approach, if language is envisioned as a system based on abstract symbols, linguistic processing needs to be able to handle abstract amodal mental representations [e.g.; 19–23]. Conversely, according to the “embodied language” processing theory, meaning depends on the brain and the body’s interactions with the social and physical environment [24–26]. Finally, the distributed language theory stresses “the centrality of coacting agents who extend their worlds and their own agency through embodied, embedded processes of languaging behavior rather than uses of an abstract language system” [27, see also 28].

These perspectives seem to have blurred the boundaries between human and non-human language converging to seemingly embrace a biologically grounded definition of biocommunication. According to this view, language can be defined as a natural system of arbitrary symbols that are generated and used following syntactic, semantic and pragmatic rules that cannot be reduced [5], and communication processing can be defined as sign-mediated, situation-appropriate interactions. Using those definitions as our starting point, we thought it would be interesting to identify the properties of language shared by different animal species and even different natural kingdoms. The literature presents several examples of parallelisms between human language and communication in non-human animal species. With regard to the combinatorial structure of human language, for example, some birds use their vocalizing apparatus (just as humans do) to produce a wide range of sound units to form complex songs. These small units in birdcalls are called syllables; ordered sets of syllables form song phrases, and phrases are then combined in various ways depending on the type of song [29].

As far as meaning-making activity is concerned, morphemes, intended as meaningful combinations of sounds, generate meaning permitting communication. Meaning-making activity takes place during a communicative exchange and is grounded in interactivity with the environment [27]. Indeed, in humans, the production of sounds for meaning- making activity stems from a social context, while attributing meaning to these sound units stems from the experience of the agents derived from their interaction with the environment [27].

Meaning-making activity, which can be thought of as a tool for sharing information to reduce uncertainty and improve survival, can also refer to animals. Communicating danger, the presence of a predator, or the amount of resources that are available are examples of meaning-making activities that can be expressed in widely different ways. For instance, monkeys use different alarm calls to warn one another depending on the classification of the predator [13,30]. But it is the waggle dance of honeybees that represents the most striking type of creative, non-stereotyped language within the animal kingdom. Through their dance, bees, are able to exchange messages and convey meanings concerning the smell, color, shape … of food sources [31]. Just as humans, bees apparently communicate about things that are beyond immediate temporal and spatial contiguity. As this creative aspect of language is present in an animal species so different from humans, the question arises: are there any “linguistic” properties that could be shared by human, animal and even brainless organisms such as plants?.

The third linguistic element considered here, dialect, can be defined as a variation of a language that is used by a particular group or population linked to geographical or social differences. Interestingly enough, there is evidence that some animal species utilize something similar to regional accents or dialects [32]. Vocal dialects have in fact been identified in the echolocation signals of some: bats [33,34], marine mammals [35], pinnipeds [36] such as the elephant seals [37] and cetacean species [38,39] such as sperm and killer whales [40,41].

In the light of these considerations and following this train of thought, a number of non-human systems seem to share some features of human language. Several animal studies have uncovered a complex communication system that has evolved without large vocabularies [31,42,43]. Moreover, an increasing number of communication codes of a variety of animal species have been broken [31,44,45]. Importantly, language is also non-verbal (e.g., gestures or facial mimicry), and in fact it is well known that animals use nonverbal forms of communication including gestures, body language, facial expressions as well as tactile, visual and chemical forms of communication [e.g., bacteria; 46–48]. Indeed, many animal species use coordinated movements to express their intentions. As explained above, honeybees share qualitative and quantitative food-related information via their waggle dance [31].

## Communication in plants

Plants are sexile organisms meaning that they are rooted to the ground in a fixed position from which they interact with the soil, other nearby plants, micro-organisms and the environment to address the challenges of obtaining adequate nutrients essential for growth. In evolutionary terms, this could explain why plants have needed to develop mechanisms to interact with their own kin and non/kin and other species to promote survival [49]. According to recently published works, two fundamental features seem to characterize plant communication: they are able to discriminate between self and non-self and to recognize kin from strangers.

With regard to the former, several studies have been able to demonstrate that plants are able to recognize their self from non-self [50] by showing, for example, that they avoid self-fertilization [51–53], which would potentially lead to weakening of the species. In order to avoid self-fertilization, the female reproductive organ, the pistil, is able to distinguish between “self-pollen,” which is rejected, and “non self-pollen,” which is accepted for fertilization [52,53]. With regard to recognizing kin from strangers, according to the kin selection theory, individuals increase their inclusive fitness via behavior that benefits the fitness of related individuals [54].

Kin selection is also linked to and made possible by kin recognition, which allows organisms to favor relatives over strangers [55]. Kin recognition in plants has been described by a number of studies [e.g., 56, 57]. 56, in fact, demonstrated that root allocation to the *Cakile edentula* plant was increased when groups of strangers shared a common pot, but not when groups of siblings shared one. The finding is in keeping with the hypothesis that greater root allocation increases the plant’s below-ground competitive ability.

If we assume that being able to discriminate between self and non-self and to recognize kin from non-kin are necessary to be able to communicate, these conditions seem to be satisfied by the plant kingdom. Additionally, the argument has been made that communication *is* essential for life and survival [5,58–63], and this goes for plants as well [61]. Plants *need* to communicate to improve their possibility of survival. But how exactly do plants communicate?

## The ways plants communicate

Clearly, plants cannot speak, but signaling between plants has been reported by some investigators who have hypothesized that plants can communicate below-ground through their root systems and above-ground via airborne and visual signals [49,64–66].

Researchers have demonstrated that plants communicate via volatile organic compounds (VOCs), a chemical “language” used to send messages by encoding them with a single scented “word” that “conveys multiple meanings depending on the intended recipients” [64]. It has also been reported that some plants add a tiny amount of nicotine to their volatiles to discourage unwanted visitors such as nectar thieves [67]; others emit methyl jasmonate (MeJA) when they are under attack.

VOCs are organic chemicals that plants release for a variety of reasons such as to respond to a predators’ attack [68–71], to attract pollinators [71], to communicate with one another [72], and to adapt to environmental stress [73]. Among their many functions, VOCs seem to play a primary role mediating plants’ above- and below-ground interactions with other organisms. Although these subtle odors are more meaningful and effective among kin [genetically identical or related plants; 57], stranger plants seem to like to “eavesdrop” and to use the information gained to implement tactical responses beneficial to their own survival [74–76].

## Volatile organic compounds (VOCs)

All plants emit their assimilated carbon into the atmosphere in the form of VOCs, which include alkanes, alkenes, alcohols, aldehydes, eters, esters and carboxylic acids [77]. While some VOCs play a role in plants’ defense and communication, the purpose of others are unknown [77].

Terpenoids, which are the most diverse of these classes of compounds, contain an integral number of 5 C carbon units, which are common to all plants, and are involved in both internal and external communication and plant defense. Terpenoids are emitted in response to internal and external factors and their information or effects are perceived by other parts of the plant itself, as well as, by other plants, animals and/or microorganisms [78]. Some plant terpenoids play a role in mediating numerous kinds of ecological interactions making them important chemical agents in plant communication [69]. Importantly from ecological and biological points of view, the concentration of terpenoids varies depending on the type of message to be conveyed. Insects are, for example, attracted by terpenoids only when low concentrations are emitted. At higher concentrations, terpenoids become more repellent [79] and less attractive to pollinators. It is indeed interesting that the ecological interactions and the effectiveness of terpenoids are dosage-dependent.

## The combinatorial aspect of language

Linguistic theories recognize the possibility to combine, in a hierarchical way, the smallest units into more structured ones in order to create new meanings as a crucial feature of language. If we apply this concept to plants, we find a parallelism with basic 5- carbon units which, in different combinations, are able to produce a variety of volatile chemical compounds to express different meanings. There is an astonishing array of structures, or terpenoid “words”, that can result from the sequential combination of its basic five-carbon units [78]. Some of these terpenoid “words” are common to all plants. It may be possible to codify the terpenoids used to form a specific message if we investigate what they emit in different environmental situations, such as in the case of danger linked to a sudden drought. In other words, if we know the meaning of a specific terpenoid and how it combines with other “chemical units” it might be possible to apprehend how a complete informative message is formed.

Terpenoids might be the key to plants’ ability to communicate meanings, as these small chemical units seem to combine to form “words” expressing various messages depending on the situation. It is known to date that a wide diversity of VOCs activated by numerous biotic and abiotic sources permits plants to use their chemical language to disseminate information efficiently. 80, for example, drew attention to the fact that plant language is endowed with true combinatorial flexibility, which means that new meanings can be assigned to old chemical words and used in new contexts leading to novel interactions. As well as having a role in attracting the natural enemies of herbivores, inducible VOCs are also used in plant-to plant signaling, pathogen defense and ozone quenching, as well as tropospheric ozone and fine-particle aerosol formation. In evolutionary terms, it was precisely through use and experience in a variety of circumstances that the inventory and the different combinations of chemical utterances were enriched with meaning and shared across generations [64].

## Meaning-making activity

How do plants assign meanings to these combinations? In humans, the symbolic units at the core of the meaning-making activity are linked to the social context, and meaning attribution of phenomenal experiences depends on the agents’ interactions with an ever-changing environment [27,81]. In the same way plant communication seems to be strictly tied to its context.

One of plants’ meaning-making activities refers to the ability to detect and decode molecule combinations containing a meaning that is critical for their survival; this is done by ignoring “non-meaningful” ones, such as those linked to pollution, animal exhalation, and artificial compounds. In addition, the activity refers to the ability to produce and understand messages of interest assigning meaning to a specific “chemical word” on the basis of interactions with the environment and the agents residing there. Orchids can provide an example of this process: in fact, some orchid species (*E. helleborine* and *E. purpurata*) release a spectrum of VOCs that are similar to those emitted by other plants when appealing for help during a predatory attack by insects such as caterpillars [82,83]. This chemical mimicry includes different kinds of volatiles, mostly six-carbon aldehydes, alcohols, acetates and other VOCs, commonly emitted by some green plants infested by herbivores [83]. Interestingly, it has been observed that some orchid species emit these specific volatile compounds in the absence of attacks by herbivores simply to attract prey-hunting social wasps for pollination. In addition, another orchid species (*Dendrobium sinense*) mimics the alarm pheromone of honeybees in order to attract wasps and hornets for pollination [82].

The two examples outlined above show that plants have the ability to mimic chemical cues, using a “chemical sign” to attract an insect of interest. Logically, this meaning-making activity is not based on representations of an idea as we are dealing with brainless systems which are unable to create representations in cognitive terms; it is rather the result of the plant’s interaction with the environment. In the case of the orchid, it is based on an interaction with and an attunement to organism of interest. An evolutionary process shaping the property across taxa and evolving the ability of making use of meanings in different forms seems to be at work here. This evolutionary process does not make the grammar and the rules behind the structure of the chemical compounds any less creative or arbitrary.

## Dialect

As 84,pointed out, language is shaped by its social and cultural context. In fact, specific environmental and social conditions determine the use and generation of signs. Dialect can thus be defined as a variation of a language used by a particular group of individuals belonging to the same ecological niche.

In the meantime, botanists have been studying the numerosity and variability of the terpenoid compounds produced and used by plants. Just as the chemical composition of plants differs widely, it is not surprising that there are wide qualitative and quantitative differences in the chemical composition of volatiles [85]. Takabayashi and colleagues [85] have studied variations in the combinations and structures of terpenoids that seem to be characteristic of a family or species. The ratio of different constituents in the emitted mixtures seems to have important ecological implications [86]. Some terpenoid mixtures may, for example, minimize the resistance of herbivores and hence delay plant defense [87–89] while others increase the potential for attracting pollinators [90].

Terpenoids are emitted in response to internal (genetic and biochemical) and external (ecological) factors and seem to have dosage-dependent effects [86]. They also vary qualitatively and quantitatively intraspecifically [85,86]. An example of this can be found in orchids pollinated by euglossine bees. To date more than 50 different volatile compounds emitted by orchids have been identified; each species of orchid has a distinct blend, and the relationships between the bees and the orchids are often highly specific [91].

Allelopathy, which is the biological phenomenon by which an organism produces one or more biochemicals affecting the germination, growth, survival and reproduction of other organisms, has been receiving increasing attention. When allelochemicals are adaptive to both the emitter and the receiver, they are classified as synomones [92]. The so-called herbivore-induced plant volatiles (HIPVs) consist of odors released by attacked plants that serve as important cues for predators of herbivorous insects to locate their prey [93]. Many volatile synomones consist of terpenoids [70,94]. Each plant species and cultivar produces its own characteristic combination of synomones induced by herbivores which means that predators come across different synomones depending on the diversity of the host plant species [85].

An interesting study examining population-specific emissions of VOCs was recently designed and conducted [75]. Going on the assumption that plants respond to volatile cues emitted by damaged neighbors to increase their defense against herbivores, some investigators attempted to determine if plant communication is more effective if it is carried out with local with respect to distant neighbors [75]. Some investigators, in fact, reported that sagebrush tissues responded to the volatile cues emitted by experimentally damaged neighboring plants to increase their levels of resistance to herbivory [75,95]. Branches incubated with the volatile cues of clipped neighbors experienced reduced levels of chewing damage compared to branches incubated with ambient air. When the investigators investigated the damaged plants, they found that sagebrush branches responded to cues from local plants from the same population more effectively than from plants originally grown 230 km away. This population-specific effect was found in both of the sites where the experiments were conducted showing that cues vary geographically in their effectiveness and suggesting that sagebrush has a stronger response to local than to foreign dialects. The investigators also observed that the volatiles emitted by damaged sagebrush plants were characterized into two heritable chemotypes (dominated by either thujone or camphor) and that following leaf damage, individuals of the same chemotype communicated more effectively than individuals of differing chemotypes [96,97]. These findings seem to indicate that chemotypes can be considered examples of language differences based on relatedness, suggesting that language is shaped by the context in which it is used and in which it develops [84].

Moreira and colleagues [97] also produced interesting results regarding plant communication characterized by geographical differences. Those investigators reported that lima bean *(Phaseolus lunatus)* plants exposed to VOCs of experimentally-damaged neighbors suffered less leaf damage than those exposed to undamaged plants, but only when neighboring plants were from the same geographical population. Additionally, they found no evidence that contrasting types of damage (a mimic of chewing herbivory and sap feeding by aphids) altered plant communication. Overall, these results suggest that plants do indeed communicate with population-specific “dialects” and that some variations in language depend on their relatedness.

## The importance of using a comparative approach to investigate plant communication

Linguists generally consider the human language the most complex one characterized by compositionality and creativity; some cultural features such as dialects are even now considered highly specific to human language. Some zoologists have recently suggested that those properties can also be found in animal communication. The fact that some of these characteristics have also been found in plants sheds new light on the ability of brainless organisms [i.e., bacteria, protozoa, fungi; 46–48, 98–101] to perceive and to communicate. These findings also imply that there is a universal process underlying communication that could explain the evolution of communicative languages. Is this the “Rosetta Stone” that could help to decode the volatile language of plants?

An important consideration regarding VOCs and plant communication presently being ventilated by some investigators is that warmer climates disturb the intricate communication system used by plants, which relies heavily on environmental variables such as the temperature. A few decades ago, it was reported that higher levels of atmospheric carbon dioxide, ozone, and temperatures seem to alter plant VOCs [102]. As Penuelas and Llusià [103] pointed out, VOCs could protect plants against high temperatures in a process linked to photorespiration. Kuokkanen and colleagues [104] reported decreased levels of flavanol glycosides and phenolics when temperatures were higher. Terpenoids were found to be affected by elevated temperatures in both spruce (*Picea abies*) and pine (*Pinus sylvestris*) [105].

Global warming and climate change have been modifying the vital signs of the planet and having devastating effects on ecosystems. Some of the most important effects of global warming on plants include modifying physiological processes such as respiration and photosynthesis, growth, development, mortality [106]. Likewise, if warming exacerbates drought conditions, plants may be less capable of coping with herbivory stress [106]. Higher temperatures and other human-caused global environmental changes such as higher ambient ozone and carbon dioxide (CO2) levels, biological invasions and habitat destruction could also play important roles in plant-insect and multitrophic interactions [106].

Even slight temperature changes could lead to important effects on the tiny volatile chemical compounds that mediate the interaction of plants with other plants and microorganisms [106]. These consequences could be both species and context specific [106] and might even affect the “dialectal communication” described above. The possible effects of global warming on plant communication are yet another reminder that plants and their communication system are indispensable to the survival and the health of the entire planet and its inhabitants.

## Future direction

Plant communication is a complex research topic that is being investigated by armies of researchers working in the fields of biology and neurobiology, botany, and cognitive sciences, agricultural science, plant physiology, evolutionary biology, chemistry and even psychology. These scientists are filing reports about the informative value of volatile signals used by plants for communication. Other reports describe water soluble molecules, which are used in plant self/non-self/kin recognition processes. In any case, the communication is rule-governed and sign-mediated. Future studies should seek to investigate the under- and above- ground parts of plants in a variety of environments and the different ways that they use to communicate. .Sensor arrays and digital image acquisition of VOCs, for example, will make it possible to study the physiological and molecular mechanisms underlying plant responses. Future studies can also seek to investigate how plants’ sensory mechanisms provide information used for defense and other survival purposes. Others are being designed to systematize the composition of terpenoids in qualitative and quantitative terms in connection to interplant signaling of stress stimuli. Interestingly, despite independent evolution of complex development in animals and plants, some researchers has begun to find repeating patterns in plants that seem to parallel those in animals. Biologists, botanists etc. who have an array of state-of-the-art, sophisticated devices and techniques at their disposal will, we hope, soon be in a position to tell us more about the mechanisms that underlie plant behavior and communication [69].

## Conclusion

Primarily communicating via VOCs, plants are able to detect, produce, and decode combinations of molecules that contain information for their survival while ignoring non- meaningful” ones. Terpenoids, small chemical compounds present in all living organisms, are important mediators of ecological interactions playing a role in plant defense against herbivores, disease resistance and in plant-plant communication. Plants are undeniably but soundlessly communicating with one another even though many of the hows and whys have yet to be uncovered [107]. Some could ask what difference does it make if plants communicate. Aside from improving our knowledge about the evolutionary and ecological processes underlying the various forms of life on this planet, the implications for biotechnology and regenerative medicine just as for sustainable agricultural practices are extensive. Finally, learning more about plant communication can help to bridge a divide that has hindered a multidisciplinary approach to this subject and may provide some insight into non-human cognition [100].
